# Id1 and Id3 co-expression correlates with clinical outcome in stage III-N2 non-small cell lung cancer patients treated with definitive chemoradiotherapy

**DOI:** 10.1186/1479-5876-11-13

**Published:** 2013-01-11

**Authors:** Eduardo Castañon, Joaquim Bosch-Barrera, Inés López, Víctor Collado, Marta Moreno, José María López-Picazo, Leire Arbea, María Dolores Lozano, Alfonso Calvo, Ignacio Gil-Bazo

**Affiliations:** 1 Department of Oncology, Clínica Universidad de Navarra, 31008, Pamplona, Spain; 2 Division of Oncology, Center for Applied Medical Research (CIMA), 31008, Pamplona, Spain; 3 Department of Radiation Oncology, Clínica Universidad de Navarra, 31008, Pamplona, Spain; 4 Department of Pathology, Clínica Universidad de Navarra, 31008, Pamplona, Spain

**Keywords:** Non-small cell lung cancer, Chemoradiotherapy, Id1, Id3, Protein expression, Prognosis

## Abstract

**Background:**

Inhibitor of DNA binding 1 (Id1) and 3 (Id3) genes have been related with the inhibition of cell differentiation, cell growth promotion and tumor metastasis. Recently, Id1 has been identified as an independent prognostic factor in patients with lung adenocarcinoma, regardless of the stage. Furthermore, Id1 may confer resistance to treatment (both, radiotherapy and chemotherapy).

**Methods:**

We have studied, using monoclonal antibodies for immunohistochemistry, the Id1 and Id3 tumor epithelial expression in 17 patients with stage III-N2 non-small cell lung cancer (NSCLC) treated with definitive chemoradiotherapy.

**Results:**

Id1 expression is observed in 82.4% of the tumors, whereas Id3 expression is present in 41.2% of the samples. Interestingly, Id1 and Id3 expression are mutually correlated (R = 0.579, p = 0.015). In a subgroup analysis of patients with the most locally advanced disease (T4N2 stage), co-expression of Id1 and Id3 showed to be related with a worse overall survival (45 vs 6 months, p = 0.002). A trend towards significance for a worse progression free survival (30 vs 1 months, p = 0.219) and a lower response rate to the treatment (RR = 50% vs 87.5%, p = 0.07) were also observed.

**Conclusions:**

A correlation between Id1 and Id3 protein expression is observed. Id1 and Id3 co-expression seems associated with a poor clinical outcome in patients with locally advanced NSCLC treated with definitive chemoradiotherapy.

## Background

Lung cancer represents the first cause of cancer death among men and women in the USA and Europe. Locally advanced stage III disease accounts for 25–30% of all cases of non-small cell lung cancer (NSCLC) at onset [1]. For patients with unresectable or inoperable stage III disease, the combination of platinum-based chemotherapy and radiotherapy has been considered the standard treatment with a 5-year survival rate of 20-30% in different studies [2,3]. Recently, we have communicated our experience in the treatment of stage III-N2 NSCLC disease, with a 5-year survival rate of 23% and a median overall survival of 41 months for patients treated with definitive concurrent chemoradiotherapy [4]. The poor clinical outcome in this subset of NSCLC patients urges the identification of specific biomarkers able to predict survival, as a priority goal in lung cancer translational research. The availability of those prognostic and predictive factors may help to identify patients who would most likely benefit from the different treatment modalities available [5]. Furthermore, the characterization of novel potential molecular targets may aid to design new personalized therapies in the different subgroups of patients identified.

The Inhibitor of DNA binding/Inhibitor of differentiation (Id) family members are part of the helix-loop-helix proteins lacking a basic DNA binding domain [6]. The Id family comprises four genes in mammals: *Id1*, *Id2*, *Id3*, and *Id4*. The Id genes have been suggested to be oncofetal genes that are expressed in embryos or fetuses, downregulated or undetectable in adult tissues, and re-expressed in tumors. They play a crucial role during embryogenesis [7], but they have been also related with cancer [8]. Thus, Id proteins can contribute to tumorigenesis by inhibiting cell differentiation, stimulating proliferation and facilitating tumor neoangiogenesis [8]. Moreover, Id1 has been shown to mediate chemotherapy resistance in hormone-independent prostate cancer cells [9,10] and ERK activation, and JNK and p38MAPK inhibition by Id1 in those cells has been suggested to be responsible for that resistance [9,10].

The prognostic role of the expression of Id family proteins in lung cancer was initially explored in small-cell lung cancer [11]. More recently, our group has studied the expression of Id1 in a large series including more than four hundred NSCLC patients. We described that Id1 expression was a poor prognostic marker for lung adenocarcinoma [12]. Thus, in radically treated stages I to III patients and stage IV patients treated with chemotherapy, higher Id1 levels were associated with a shorter disease-free survival and overall survival in adenocarcinoma patients. Moreover, a Cox model confirmed the independent prognostic value of Id1 levels for these patients. More interestingly, we showed that Id1 downregulation in primary treatment-resistant adenocarcinoma cell lines sensitizes those cells to radiotherapy and chemotherapy treatment *in vitro*[12], although no mechanistic experiments where performed to explain that observation. In other neoplasms, such as prostate cancer, the overexpression of Id1 has been also related to radioresistance [13].

Id1 and Id3 are considered to have overlapping and synergistic functions in cancer biology and have been related with the inhibition of cell differentiation, cell growth promotion and tumor metastasis [14]. To our knowledge, the Id3 protein expression in human NSCLC has never been explored, nor its correlation with Id1 expression or its predictive value.

In the present manuscript, we have studied the potential prognostic and predictive role of Id1 and Id3 expression by immunohistochemistry in stage III-N2 patients treated with definitive chemoradiotherapy.

## Methods

### Patients’ characteristics and tissue samples

We explored a cohort of 34 patients with stage III-N2 NSCLC disease treated, as previously published [4], with definitive concurrent chemoradiotherapy and followed at the Clínica Universidad de Navarra (CUN) between 1996 and 2006.

These patients were radically treated with induction platinum-based chemotherapy, followed by concurrent chemotherapy and hyperfractioned 3D-CRT [1.2 Gy b.i.d.; median dose: 66.5 Gy (range: 64–74)]. Paclitaxel (50 mg/m2) and cisplatin (30 mg/m2) were administered intravenously over a 60-min period on days 1, 8, 15, 22, 29 and 36 after confirmation of adequate blood cells counts.

From the whole cohort, tumor tissue samples were available and evaluable by immunohistochemistry for Id1 and Id3 expression in 17 patients. In 15 of the cases, samples represented primary tumor tissues and in remaining 2, lymph node biopsies were included assuming a homogeneous Id1 and Id3 expression between primary and lymph node samples. Of these 17 patients 70.6% were male; the median age of the cohort was 54 years (range, 41–78 years); 76.5% of the tumors showed squamous cell histology, 11.8% showed adenocarcinoma and 11.7% were NSCLC not otherwise specified (NOS). The study protocol was approved by the ethical committee of our institution.

### Immunohistochemical analysis

Id1 and Id3 expression was evaluated using indirect immunoperoxidase staining of formalin-fixed paraffin-embedded tissue sections (9 samples) as well as cell block sections (2 samples), as described previously [15]. The rabbit monoclonal anti-mouse/human Id1 (L/N: RN-42730) and Id3 (L/N: RN-38144) antibodies (1:100; Biocheck, CA, USA) were used. Detection was conducted with the AdvanceTM HRP system (Dako, Glostrup, Denmark).

In addition, alcohol-fixed cytology slides from fine-needle biopsies were the only tumor material available in six patients, and they were processed as previously described [16]. Briefly, each sample was exposed to 100% acetone for 5 min using an individual coupler per sample, to facilitate coverglass removal. During the next 5 min, samples were washed with 100% acetone and dried, After, washing samples with 100% ethanol for 3 minutes, endogenous peroxidase activity was blocked by incubation in 3% hydrogen peroxide in methanol for 30 min in a dark room, and then the samples were rinsed with 100% ethanol for 3 minutes followed by sequential washes in decreasing concentrations of ethanol. After this point, samples followed the standard protocol for biopsy tissues.

Staining scores were determined by a semiquantitative analysis (H-score) as previously described [17]. Briefly, the extension and intensity of the staining was evaluated by two observers, independently and blinded for any clinical data. The extension was scored as a percentage of positive cells (0 = 0%, 1 = 1-5%, 2 = 5-50% and 3 = 50-100%) and the intensity of staining was assessed compared with a known external positive control (0 = no staining, 1 = weak, 2 = moderate, and 3 = strong staining). A final H-score was calculated using the products of the percentage of cells stained at a given staining intensity (0–3) and the staining intensity (0–3). The expression was analyzed in the tumor epithelial cells. Staining of vascular endothelial cells for Id1 and Id3 served as an internal control for immunohistochemistry when possible (tumor biopsies) as previously published [12]. In immunostaining analysis with cell blocks and tumor cytology smears, samples were run together with known positive samples used as positive controls.

### Statistical analysis

The correlation between Id1 and Id3 H-score was evaluated by the Spearman’s rank correlation coefficient. This non parametric test was also applied to analyze the relation between the product of Id1 and Id3 H-score and the overall survival (OS) and progression-free survival (PFS). The relation between Id1 and Id3 expression and the response rate was also studied by using the MacNemar’s test. Kaplan–Meier survival curves were generated to evaluate the PFS and OS. A log-rank test was performed to find statistical differences in the Kaplan–Meier survival analyses. Statistical significance was defined as p-values < 0.05. The SPSS 15.0 software (SPSS, Inc., Chicago, IL) was employed to perform the statistical analysis.

## Results

### Expression of Id1 and Id3 in NSCLC tissue by immunohistochemistry

**Figure 1:**
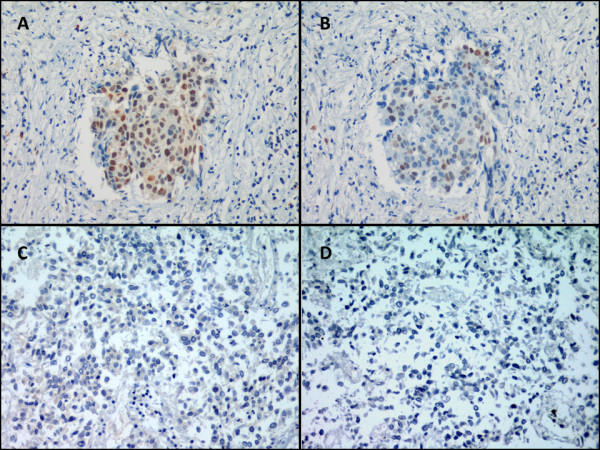
**Id1 and Id3 immunohistochemistry in non-small cell lung cancer samples.** Id1 expression (20×) with final H-score of 9 (**A**) and Id3 expression (20×) with final H-score of 6 (**B**). Lack of Id1 (20×) (**C**) and Id3 (20×) (**D**) expression with final H-score of 0.

Tumor samples from 17 patients out of the 34 NSCLC patients treated with definitive chemoradiotherapy were available. Clinical characteristics of the 17 patients analyzed are summarized in table 1. The tumor epithelial expression and H-score of Id1 and Id3 of these patients is detailed in table 2. Id1 expression was observed in 82.4% of the patients, whereas Id3 expression was present in the 41.2% of the patients (figure 1). All patients that presented Id3 expression had also Id1 expression. We were able to identify Id1 and Id3 epithelial expression in all kinds of samples available (biopsy, cell block, and cytology).

**Table 1:** Clinical characteristics of the patients analyzed

**Gender**	**Number**	**(%)**
**Male**	12	70.6%
**Female**	5	29.4%
**Histology**	**Number**	**(%)**
**Adenocarcinoma**	2	11.8%
**Squamous cell**	13	76.4%
**Other**	2	11.8%
**Initial cT**	**Number**	**(%)**
**cT1**	1	5.9%
**cT2**	3	17.6%
**cT3**	4	23.5%
**cT4**	9	53%
**Age (years)**	**Median**	**Range**
	54	41 – 78

**Table 2:** Pattern of expression of Id1 and Id3 proteins in the 17 evaluable patients

**Patient number**	**Tissue**	**Staining intensity**		**Percentage of staining**		**H-score**	
		**Id1**	**Id3**	**Id1**	**Id3**	**Id1**	**Id3**
10	LB	1	0	2	0	2	0
2	BB	2	1	3	1	6	1
3	BB	2	2	1	1	2	2
5	BB	1	1	1	1	1	1
6	BB	1	1	2	1	2	1
7	BB	2	1	2	2	4	2
8	BB	1	0	3	0	3	0
1	LNB	3	3	3	2	9	6
11	LNB	0	0	0	0	0	0
4	CB	0	0	0	0	0	0
9	CB	1	0	2	0	2	0
12	C	0	0	0	0	0	0
13	C	1	0	2	0	2	0
14	C	0	0	0	0	0	0
15	C	1	0	2	0	2	0
16	C	1	0	1	0	1	0
17	C	1	1	2	1	2	1

Tissue sample: *LB* (lung biopsy), *BB* (bronchial biopsy), *LNB* (lymph node biopsy), *CB* (cell block sample), *C* (cytology sample). Staining intensity (0: no staining, 1: weak, 2: moderate, 3: strong staining). Percentage of staining (0 = 0%, 1 = 0-5%, 2 = 5-50%, 3 > 50%).

### Prognostic value of Id1 and Id3 expression

**Figure 2:**
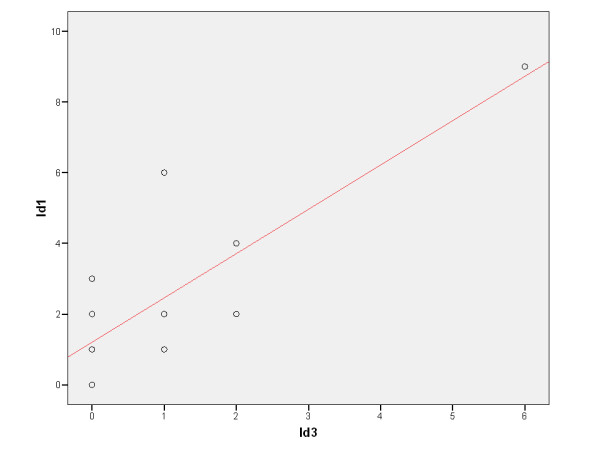
Spearman’s rank correlation curve between Id1 and Id3 in all patients with tumor sample available for the study (n = 17).

We investigated whether the expression of Id1 and Id3 in stage III-N2 NSCLC patients treated with definitive chemoradiotherapy could serve as a prognostic biomarker. First, we observed that the expression of Id1 and Id3 were significantly correlated in a positive trend (R = 0.579, p = 0.015), (figure 1, A and B; figure 2).

For this reason and considering the well-known synergies between both Id genes, we hypothesized that the product of the H-score of Id1 and Id3 (Id1/Id3) could better correlate with the clinical outcomes to the treatment in the specific subgroup of patients with more advanced stage (cT4N2 disease) treated with definitive concurrent chemoradiotherapy (n = 9).

Firstly, the response rate (RR) achieved with the treatment seemed to be lower among patients showing a tumor Id1/Id3 co-expression compared to those showing no Id1/1d3 co-expression with a trend towards statistical significance using the MacNemar’s test, (RR = 50% vs 87.5%, respectively; p = 0.07). Accordingly, a significant correlation between Id1/Id3 co-expression and the OS (R = −0.733, p = 0.025) was observed. The correlation between Id1/Id3 co-expression and the PFS also showed a trend towards the statistical significance (R = −0.639, p = 0.064).

**Figure 3:**
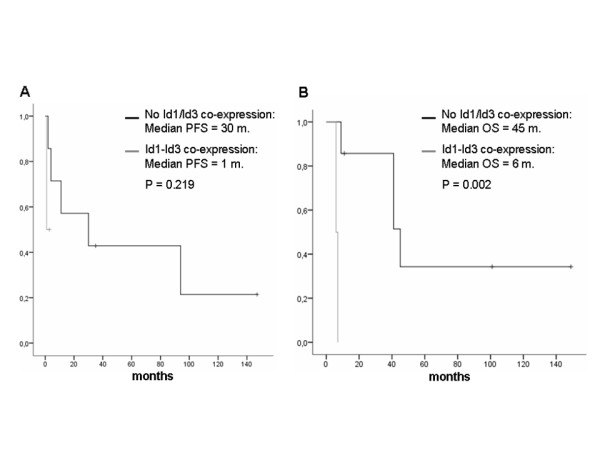
Kaplan-Meier curves for Progression-Free Survival (PFS) (A) and Overall Survival (OS) (B) of T4N2 patients with or without Id1/Id3 co-expression.

These results were confirmed by the Kaplan-Meier curves and log-rank test. A significantly worse prognosis in terms of OS for patients that presented co-expression of Id1/Id3 in their tumor samples compared to those with a complete lack of Id1/Id3 co-expression (45 months vs 6 months; p = 0.002), was observed, as showed in figure 3. PFS also showed differences in favor of patients showing no Id1/Id3 tumor co-expression although a statistical significance was not achieved (30 months vs 1 month, p = 0.219), (figure 3). However, for those patients with tumor samples showing an exclusive Id1 expression in the absence of Id3 expression, no impact of that expression on clinical outcomes was observed. In fact, the OS for patients with Id1-expressing tumors was 45 months compared to 41 months in subjects with tumors showing no Id1 expression, p = 0.646. Similar results were obtained for PFS (94 months versus 11 months respectively, p = 0.588).

Finally, no correlation between the exclusive expression of Id3 in the absence of Id1 expression with clinical outcomes could be studied because in our series, all patients with Id3-expressing tumors concurrently showed Id1 expression, as shown in table 2.

## Discussion

In a previous study, we showed that a high Id1 protein expression is an independent prognostic biomarker in patients with adenocarcinoma of the lung, regardless the stage [12]. Also, we observed that Id1 silencing may sensitize adenocarcinoma cells to radiotherapy and chemotherapy [12].

In the present study we tried to validate the previous *in vitro* observations in a clinical series of NSCLC patients receiving chemoradiotherapy with radical intent. Despite some limitations regarding its retrospective nature and the relatively short cohort studied, we find for the first time that the co-expression of Id1 and Id3 in the tumor epithelium can be a powerful prognostic biomarker in a selected subgroup of stage III-N2 NSCLC patients receiving definitive chemoradiotherapy.

In several studies with neuroblastoma cell lines, Id genes have been shown to be overexpressed by tumor hypoxia [18,19]. On the other hand, in several tumor types, tumor hypoxia has been well characterized as a key factor in the induction of radioresistance [20,21]. Thus, it seems biologically plausible that higher Id1 and Id3 tumor expression levels may reflect the hypoxic status of the neoplasm and therefore would explain the radioresistance observed in those patients. In fact, elevated Id1 and Id3 protein levels have been found *in vitro* and by immunohistochemistry in samples of many different human carcinoma types supporting the role of both genes in carcinogenesis [8].

The correlation between Id1 and Id3 and its functional implications have been studied in other cancers. For example, different breast tumors failed to grow and/or metastasize in Id1 (+/−) Id3 (−/−) knockout mice [22]. In addition, when Id1 and Id3 are both interfered by Id1/3-PA7, an antiproliferative and apoptotic effect can be observed in breast cancer cells [22]. Similarly, the combined inhibition of Id1 and Id3 in human pancreatic tumor cells resulted in decreased ability of pancreatic cancer cells to proliferate and migrate. In addition, Id1/Id3 double-knockdown caused decreased expression of integrins alpha3, alpha6, and beta1, and consequently reduced adhesion of tumor cells to laminin. Finally, peritoneal metastases of Id1/Id3 double-knockdown tumor cells were significantly reduced in a mouse model of peritoneal metastasis [23]. Moreover, functional studies have shown that Id1 and Id3 are required for both, tumor initiation and during metastatic colonization of the lung microenvironment by breast cancer cells [24]. Recently, Id1 and Id3 co-expression (but not its individual expression) has been proposed as a novel mechanism of regulation of the self-renewal capacity of human colon cancer-initiating cells (CC-ICs) [25]. In that study, regulation of p21 exerted by Id1 and Id3 seemed to be a central mechanism preventing the accumulation of excess DNA damage and subsequent functional exhaustion of CC-ICs. Remarkably, the Id1 and Id3 co-silencing in LS174T colon cancer cells in the same study, was able to increase the sensitivity of colon cancer-initiating cells to oxaliplatin, linking tumor initiation with chemotherapy resistance and underlying the potentially synergistic properties between Id1 and Id3 [25]. Furthermore, in a study by Perry et al., Id1 seemed to have a role in long-term repopulating hematopoietic stem-cell maintenance and hematopoietic development, and more interestingly, it was able to functionally compensate the Id3 loss presumably due to its similar biological properties [26]. All these evidences seem to indicate that although Id1 and Id3 may show some similar and redundant biological properties during embryonic development and tumorigenesis, their differential regulation could explain their complementary and ultimately distinct functions, as demonstrated in the postmitotic Sertoli cell [27].

With regard to NSCLC, other authors have also observed that Id1 is expressed in a nuclear pattern in the majority of squamous cell carcinomas (70%) and non-squamous cell carcinomas (50%) of the lung [17]. However, the expression and potential role of Id3 and its correlation with Id1 expression have not been previously studied in NSCLC patients.

In the present study, also for the first time, tumor cell blocks and cytology smears have been included for Id1 and Id3 immunostaining analysis. This approach represents a challenge but could also implicate a potential methodological caveat due to the small quantity of tissue that may underestimate the expression of both proteins. However, in the last several years, especially in the field of lung cancer, immunocytochemestry has gained importance in better characterizing samples with diagnostic and prognostic purposes showing a reliable performance for most of the antibodies used [28].

Although the expression of Id1 and Id3 were mutually correlated in a positive trend in our series, a remarkably higher (and more frequent) expression of Id1 compared to Id3 was observed in the NSCLC samples analyzed. This represents a novel finding since no other study has previously investigated the expression of both proteins in the same NSCLC samples. Whether this more intense and abundant Id1 expression among NSCLC samples, compared to Id3 expression, is able to functionally compensate a lower expression of Id3 warrants a further *in vitro* and *in vivo* investigation. More interestingly, the co-expression of both proteins Id1 and Id3, in the same tumor samples, showed a significant inverse correlation with OS in those patients showing a clinical stage considered of worse prognosis (cT4N2 disease). Those results were also corroborated by the Kaplan-Meier and log-rank tests showing a significantly worse prognosis in terms of OS for patients that presented co-expression of Id1/Id3 in their tumor samples compared to those with a complete lack of Id1/Id3 co-expression (45 months vs 6 months; p = 0.002). Additionally, the PFS analysis showed similar differences in favor of patients lacking Id1/Id3 co-expression in their tumor samples, although the limited number of patients included could have accounted for the lack of statistical significance observed. Finally and according to our previous results *in vitro*, the response rate associated with the chemoradiotherapy administered to the patients was a 37.5% lower among those subjects with tumors showing a co-expression of Id1 and Id3. However, this observation only showed a trend towards significance probably due to insufficient statistical power.

An isolated Id1 expression had no significant impact on the clinical outcome in our patients possibly due to the limited number of patients included in this cohort. However, this result could also be justified by the fact that the most frequent histology in our series was squamous cell carcinoma (76.4%), and, as previously published, Id1 expression has no prognostic impact among patients bearing tumors with this histology [12].

## Conclusions

In conclusion, the present study suggests that the tumor co-expression of Id1 and Id3 can be a powerful prognostic biomarker in a selected subgroup of stage III-N2 NSCLC patients receiving definitive chemoradiotherapy.

Further prospective studies in larger patient cohorts are warranted to better define the exact prognostic significance of the Id1/Id3 co-expression in NSCLC and whether the isolated expression of one or the other is sufficient to observe the same effect. A comparison of Id1 and Id3 expression between primary tumor and matching metastatic tissues should be also investigated. At the same time, the subjacent molecular mechanisms involved, such as the tumor hypoxia, should be prospectively explored, especially in patients treated with chemoradiotherapy, in whom these proteins might play a role in the resistance of tumor cells to the different multimodal therapeutic strategies.

## Abbreviations

NSCLC: Non-small cell lung cancer; Id: Inhibitor of DNA binding/Inhibitor of differentiation; CUN: Clínica Universidad de Navarra; PFS: Progression-free survival; OS: Overall survival; RR: Response rate.

## Competing interests

The authors declare that they have no competing interests.

## Authors’ contributions

EC participated in the design of the study, contributed to the patients’ identification and medical history charts revision and performed immunostaining and statistical analyses. JBB participated in the design of the study, contributed to the patients’ identification and medical history charts revision and performed immunostaining and statistical analyses. IL carried out immunostaining analysis. VC contributed to the immunostaining analysis and paper drafting. MMJ contributed to drafting the manuscript and revising it critically for important intellectual content. JMLP aided to conceive the study, and participated in its design and helped to draft the manuscript. LA contributed to drafting the manuscript and revising it critically for important intellectual content. MDL selected the tumor tissue samples and contributed to the immunostaining analysis. AC contributed to drafting the manuscript and revising it critically for important intellectual content. IGB conceived the study and its design, contributed to patients’ selection, immunohistochemistry analysis, helped to draft the manuscript and gave final approval. All authors read and approved the final version of the manuscript.
